# Control of Cell Size by c-di-GMP Requires a Two-Component Signaling System in the Cyanobacterium *Anabaena* sp. Strain PCC 7120

**DOI:** 10.1128/spectrum.04228-22

**Published:** 2023-01-10

**Authors:** Qin-Xue Sun, Min Huang, Ju-Yuan Zhang, Xiaoli Zeng, Cheng-Cai Zhang

**Affiliations:** a State Key Laboratory of Freshwater Ecology and Biotechnology and Key Laboratory of Algal Biology, Institute of Hydrobiology, Chinese Academy of Sciences, Wuhan, Hubei, People’s Republic of China; b University of Chinese Academy of Sciences, Beijing, People’s Republic of China; c Institut WUT-AMU, Aix-Marseille Univ. and Wuhan University of Technology, Wuhan, Hubei, People’s Republic of China; Wuhan University

**Keywords:** cell morphology, cell size, cyanobacteria, c-di-GMP, signal transduction

## Abstract

Each bacterial species possesses a specific cell size and morphology, which constitute important and recognizable physical traits. How bacteria maintain their particular cell size and morphology remains an essential question in microbiology. Cyanobacteria are oxygen-evolving photosynthetic prokaryotes. Although monophyletic, these organisms are highly diverse in their cell morphology and cell size. How these physical traits of cyanobacteria are controlled is poorly understood. Here, we report the identification of a two-component signaling system, composed of a histidine kinase CdgK and a response regulator CdgS, involved in cell size regulation in the filamentous, heterocyst-forming cyanobacterium *Anabaena* sp. PCC 7120. Inactivation of *cdgK* or *cdgS* led to reduction of cell length and width with little effect on cell growth capacity. CdgS has a GGDEF domain responsible for the synthesis of the second messenger c-di-GMP. Based on genetic and biochemical studies, we proposed a signaling pathway initiated by CdgK, leading to the phosphorylation of CdgS, and thereby an enhanced enzymatic activity for c-di-GMP synthesis of the latter. The GGDEF domain of CdgS was essential in cell size control, and the reduction of cell size observed in various mutants could be rescued by the expression of a c-di-GMP synthetase from E. coli. These results provided evidence that a minimal threshold of c-di-GMP level was required for maintaining cell size in *Anabaena*.

**IMPORTANCE** Cyanobacteria are considered the first organisms to produce oxygen on Earth, and their activities shaped the evolution of our ecosystems. Cell size is an important trait fixed early in evolution, with the diversification of micro- and macrocyanobacterial species during the Great Oxidation Event. However, the genetic basis underlying cell size control in cyanobacteria was not understood. Our studies demonstrated that the CdgK-CdgS signaling pathway participates in the control of cell size, and their absence did not affect cell growth. CdgK has multiple domains susceptible to signal input, which are necessary for cell size regulation. This observation suggests that cell size in *Anabaena* could respond to environmental signals. These studies paved the way for genetic dissection of cell size regulation in cyanobacteria.

## INTRODUCTION

Cell size is a critical parameter for microbial morphology and varies greatly among different bacteria, even within the same species (1 to 3). Generally, each microorganism has its distinct cell size under steady-state conditions, which is maintained through the generations (4 to 7). For that reason, bacteria must coordinate cell growth, cell division, and DNA replication to control cell size, and abnormalities in any of these functions can cause changes in cell size (5, 8, 9). Although there is little variation in cell size under constant conditions, changes in environmental conditions can affect the average cell size of bacteria (10). Therefore, cells must have signal transduction pathways to perceive and transmit environmental cues to the cell-size maintaining machineries in order to govern cell size (11). The stringent response signal ppGpp has been reported to be a regulator of cell size in bacteria (12).

c-di-GMP (bis-(3-5)-cyclic dimeric GMP) emerged as one of the most common and important bacterial second messenger. It regulates various physiological processes, including biofilm formation, cell motility, virulence, cell differentiation, peptidoglycan synthesis, mechanosensing, etc. (13 to 16). The intracellular c-di-GMP level depends on two types of enzymes with opposing activities: diguanylate cyclases (DGCs) and specific phosphodiesterases (PDEs) (14 to 16). One molecule of c-di-GMP is synthesized from two molecules of GTP by DGC, and is hydrolyzed to one 5′-phosphoguanylyl-(3′–5′)-guanosine (pGpG) or two GMP by PDE. DGC activity is associated with the GGDEF motif present in the corresponding enzymes at the active site, while the PDE activity is associated with the EAL or some of the HD-GYP domains (14 to 16). Often, the GGDEF, EAL, or HD-GYP domains are found in one protein, and coupled with various sensory domains, such as CHASE (cyclase/histidine kinases associated sensing extracellular), PAS (Per–ARNT–Sim), GAF (cGMP phosphodiesterase/adenylate cyclase/FhlA), REC (receiver domain in Two-Component Signaling Systems [TCS]), etc. (16). These domains perceive the environmental cues such as oxygen (17), blue light (18), nutrient starvation (19), antibiotics (20), or intercellular chemical signaling molecules such as the state of protein phosphorylation, or mucin (16, 21). Accordingly, the intracellular c-di-GMP level can be modulated through the control of the DGC or PDE activities by various internal or external environmental signals.

A classical TCS is composed of a signal-sensing histidine kinase (HK) and a cytoplasmic cognate response regulator (RR) (22 to 24). Upon stimulation by environmental cues, HK becomes autophosphorylated at a conserved His residue. The phosphoryl group is then transferred to a conserved Asp residue in the receiver (REC) domain of the cognate RR. The phosphorylated RR modulates bacterial response to intracellular or extracellular environmental cues by controlling gene expression, enzyme activity, RNA–protein interaction, or protein–protein interaction (22 to 25). Many TCSs also use intermediary His-phosphotransferase (Hpt) domains to form a multistep phosphorelay. No matter how complex the phosphoryl group transfer between a HK and its cognate RR is, the specificity among them is high (26, 27).

*Anabaena* PCC 7120 (*Anabaena*, also known as *Nostoc* PCC 7120) is a filamentous cyanobacterium and contains 16 genes encoding proteins with a c-di-GMP synthesis or hydrolysis domain, or both (28, 29). In this organism, cells, called heterocysts, dedicated to nitrogen fixation, can be induced within 24 h upon combined-nitrogen deprivation (30). Heterocysts are intercalated among vegetative cells in a semiregular pattern, which constitutes a simple process of developmental pattern formation. A number of genes have been identified for the establishment (24 h after the induction) or the maintenance of a heterocyst pattern (48 h or longer after the induction) (30). It was reported that the inactivation of *cdgS* (*all2874*) encoding a c-di-GMP synthetase with a GGDEF domain caused a significant reduction in heterocyst frequency and in cell size of vegetative cells during diazotrophic growth compared to the wild type, and the severity of these phenotypes varied with changes in light intensity (28). Our previous study further indicated that c-di-GMP homeostasis was critical for heterocyst development. We found that in addition to CdgS, the bifunctional enzyme CdgSH, which possesses both c-di-GMP synthesis and hydrolysis activities, was also involved in the control of c-di-GMP levels during heterocyst differentiation (29).

Cell size is a physical trait fixed early during evolution, with the diversification of cyanobacterial species, and the increase of cell size during the Great Oxidation Event about 2.45 billion years ago (31). Such an innovation led to the appearance of microcyanobacterial (less than 3 μm in cell diameter) and macrocyanobacterial (3 to 50 μm in cell diameter) clades (31). The underlying genetic basis for cell size diversification remains unknown. In the unicellular cyanobacterium Synechococcus elongatus, the circadian rhythm and environment have been shown to control cell size (32). In *Anabaena*, the nitrogen and carbon regime could influence cell size, and the *mreBCD* operon regulates both cell size and morphology (33). In this study, we demonstrated that c-di-GMP plays a critical role in the control of cell size in *Anabaena*. Furthermore, we found that a two-component system, consisting of CdgK (All2875, HK) and CdgS (All2874, RR), is involved in cell size control by regulating the intracellular c-di-GMP level. Our studies paved the way for genetic dissection of the control mechanism of the cell size, a primordial physical trait that characterizes each bacterial species.

## RESULTS

### Heterologous expression of the *yhjH* gene caused a dramatic reduction in cell size of *Anabaena*.

**FIG 1:**
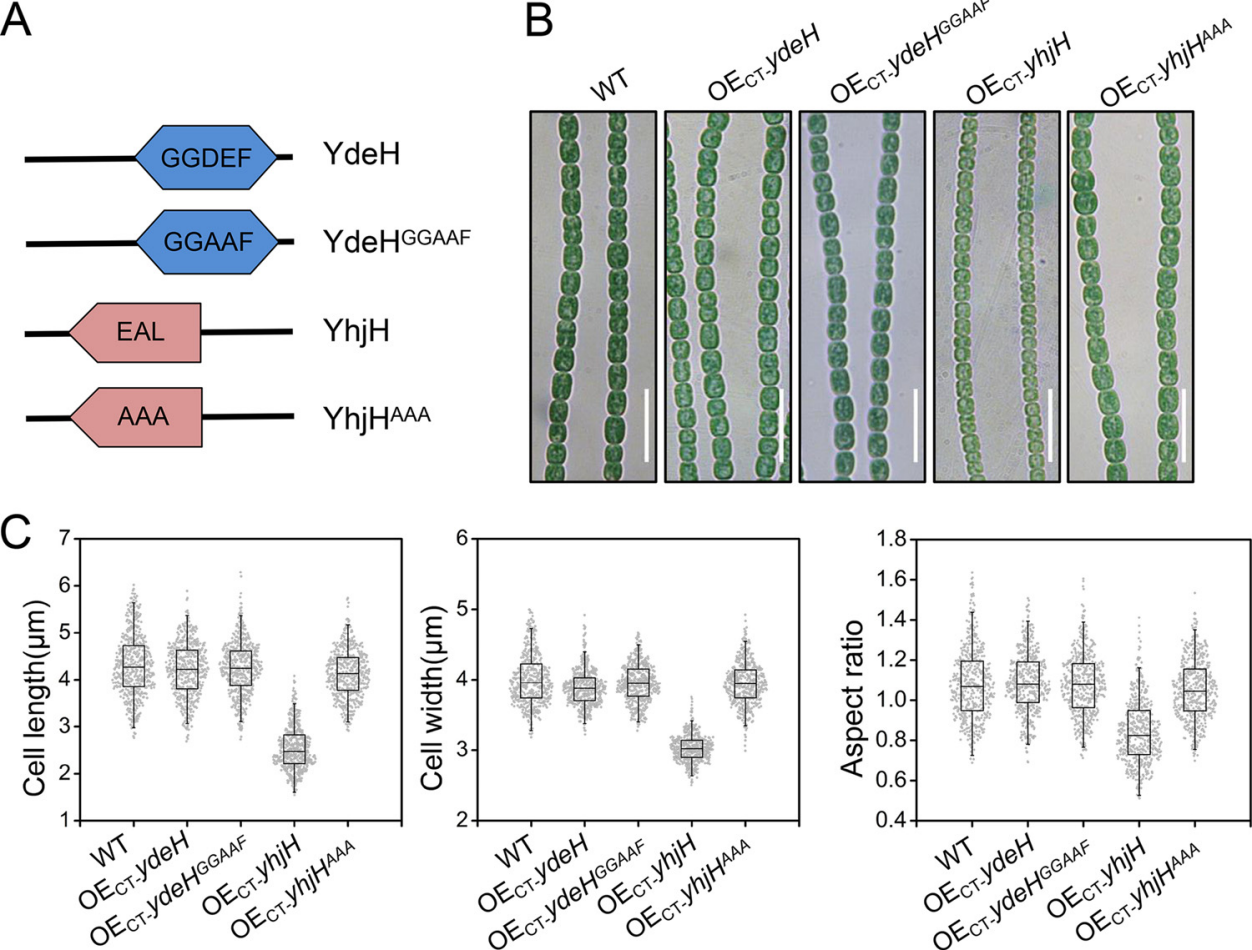
Expression of the high-efficient c-di-GMP hydrolase YhjH from E. coli in *Anabaena* affects cell size. (A) Schematic representation of YdeH, a c-di-GMP synthetase, and YhjH, a c-di-GMP hydrolase, and their corresponding inactive mutant forms from E. coli deduced by SMART (smart.embl-heidelberg.de). GGDEF, diguanylate cyclase (DGC) domain; GGAAF, inactive diguanylate cyclase domain (with E208A and E209A mutations inactivating the catalytic and the metal ion coordination sites); EAL, phosphodiesterase (PDE) domain; AAA, inactive phosphodiesterase domain (with E48A, L49A, and L50A inactivating the catalytic and the metal ion coordination sites). (B) Micrographs of *Anabaena* filaments of WT or different overexpression strains cultured in BG11 after 48 h of induction with 0.5 μM copper and 2 mM theophylline. Scale bars represent 15 μm. *ydeH*, a gene from E. coli encoding a c-di-GMP synthetase. *yhjH*, a gene from E. coli encoding a c-di-GMP hydrolase. *ydeH^GGAAF^*, a mutant expressing inactive *ydeH*. *yhjH^AAA^*, a mutant expressing an inactive form of *yhjH*. (C) Statistic analysis of cell length, cell width, and aspect ratio. Five hundred cells of each strain were measured.

*yhjH* and *ydeH*, from E. coli, encode a PDE and a DGC, respectively (Fig. 1A) (34, 35). YdeH and YhjH show strong catalytic activities and are thus used as a tool to control the intracellular c-di-GMP level in various bacteria (36, 37). Our previous study showed that after overexpression in *Anabaena*, strain OE_CT_-*ydeH* led to an elevated intracellular level of c-di-GMP, while strain OE_CT_-*yhjH* displayed a decreased intracellular level of the same secondary messenger (29). In this study, when observed under a microscope, we found that cells of the strain OE_CT_-*yhjH* displayed a cell size that was significantly smaller than that of the wild-type (WT) strain, while no particular cell size changes were observed in strain OE_CT_-*ydeH* (Fig. 1B). As controls, no change of cell size could be found in strain OE_CT_-*yhjH^AAA^* or OE_CT_-*ydeH^GGAAF^*, bearing the same plasmids but expressing an inactive form of YhjH or of YdeH, respectively (Fig. 1B). Statistical analysis of cell length, cell width, and the aspect ratio of 500 cells for each of the 5 strains further confirmed the results (Fig. 1C). The average cell length, cell width, and aspect ratio (cell length divided by cell width) were 2.5 μm, 3.0 μm, and 0.8 for the OE_CT_-*yhjH* strain, while the same parameters of other strains (WT, OE_CT_-*ydeH*, OE_CT_-*yhjH^AAA^*, and OE_CT_-*ydeH^GGAAF^*), were 4.1 μm, 3.9 μm, and 1.03, respectively (Fig. 1C). Expressed as cell volume, the OE_CT_-*yhjH* strain gave a cell volume at 18.4 ± 4.5 μm^3^, compared to 51.2 ± 9.7 μm^3^ for the WT (Table 1). Therefore, the expression of *yhjH* reduced both cell length and cell width and dramatically decreased cell volume. These results suggested that c-di-GMP could play an important role in cell size control in *Anabaena*.

**TABLE 1:** Analysis of cell volume of the wild type (WT) and different mutant strains^*a*^

Strain	Vol (μm^3^)
WT	51.2 ± 9.7
OE_CT_-*yhjH*	18.4 ± 4.5
OE_CT_-*yhjH*^AAA^	51.0 ± 10.7
OE_CT_-*ydeH*	50.4 ± 10.5
OE_CT_-*ydeH*^GGAAF^	52.3 ± 10.6
*ΔcdgS*	19.1 ± 5.2
*CcdgS*	52.7 ± 10.9
*CcdgS^GGAAF^*	21.9 ± 5.2
*ΔcdgS::ydeH*	53.2 ± 10.5
*ΔcdgS::ydeH^GGAAF^*	19.2 ± 4.2
*cdgS^GGAAF^*	22.8 ± 4.9
*cdgS^D60A^*	24.1 ± 5.5
*ΔcdgK*	22.6 ± 4.8
*CcdgK*	48.4 ± 11.3
*ΔcdgK::ydeH*	49.1 ± 9.7
*ΔcdgK::ydeH^GGAAF^*	23.5 ± 5.9
*ΔcdgKΔcdgS*	23.1 ± 5.1
*ΔcdgKΔcdgS::ydeH*	51.3 ± 12.4
*ΔcdgKΔcdgS::ydeH^GGAAF^*	22.7 ± 5.4
*cdgK^ΔTM^*	23.3 ± 5.1
*cdgK^ΔCHASE^*	21.3 ± 4.7
*cdgK^ΔPAS1^*	45.7 ± 10.4
*cdgK^ΔPAS2^*	53.6 ± 13.7
*cdgK^ΔPAS3^*	22.1 ± 4.5
*cdgK^ΔPAS4^*	22.7 ± 5.3
*cdgK^ΔPAS5^*	23.6 ± 5.4
*cdgK^ΔGAF^*	23.1 ± 4.8
*cdgK^ΔHK^*	22.6 ± 5.1
*cdgK^ΔREC1^*	52.1 ± 14.2
*cdgK^ΔREC2^*	22.9 ± 5.7
*cdgK^ΔHpt^*	22.4 ± 5.4
*cdgK^H1149A^*	22.3 ± 4.8
*cdgK^D1462A^*	49.7 ± 10.5
*cdgK^D1604A^*	22.8 ± 5.0
*cdgK^H1757A^*	23.2 ± 5.3

a All values are shown as mean ± standard deviation, calculated using the results obtained with 500 cells in a total from three biological replicates.

### *ΔcdgS* displayed a significant reduction in cell size.

**FIG 2:**
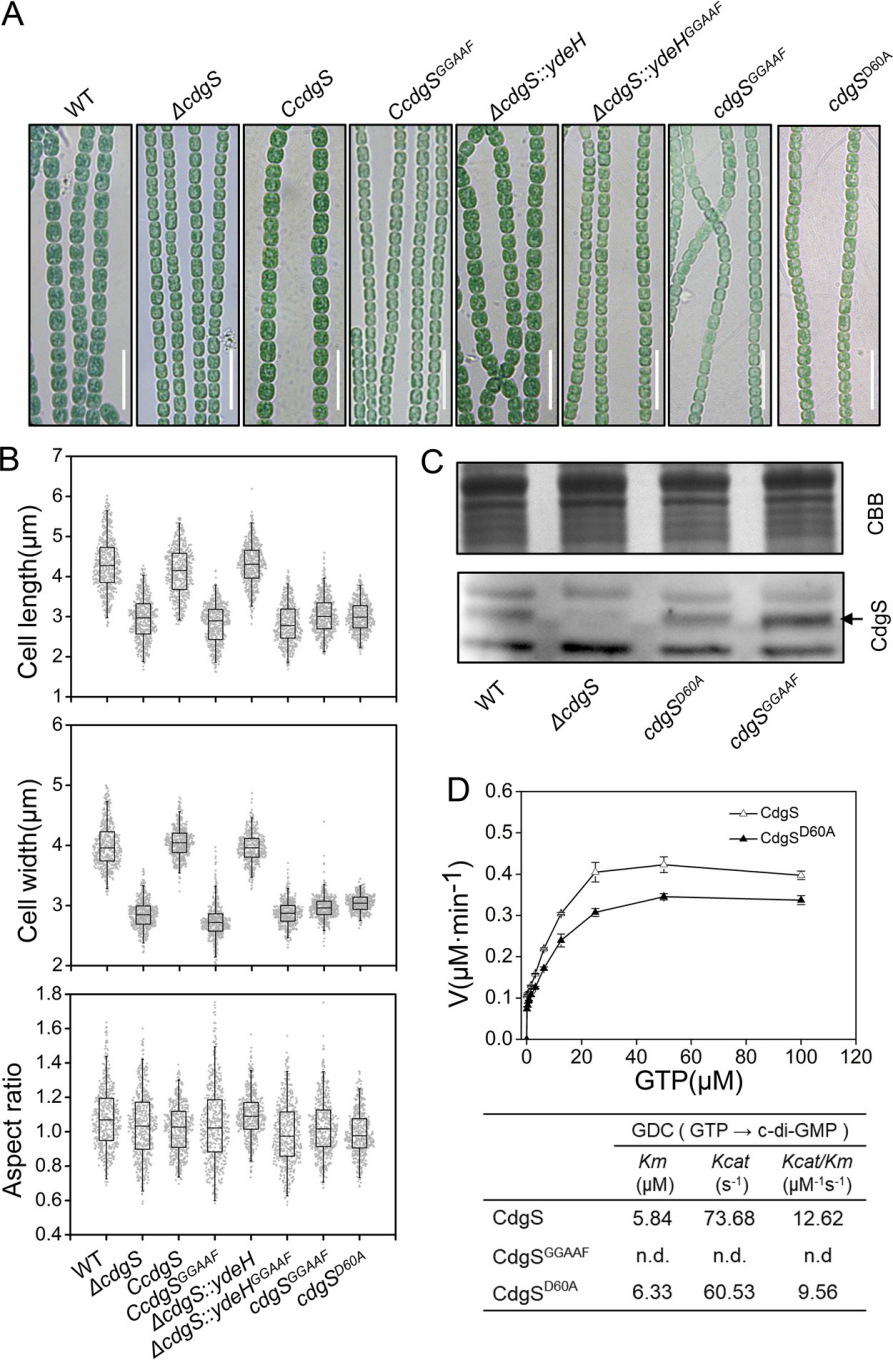
Function of c-di-GMP synthesis activity of CdgS in the control of cell size. (A and B) Microscopic observation (A) and cell size parameters (B) of WT, *ΔcdgS*, *CcdgS*, *CcdgS^GGAAF^*, *ΔcdgS::ydeH*, *ΔcdgS::ydeH^GGAAF^*, *cdgS^GGAAF^*, and *cdgS^D60A^* cultured in BG11 medium. Five hundred cells were measured for each strain. All experiments were done with three replicates. Scale bars: 15 μm. (C) Western blot of WT, *ΔcdgS*, *cdgS^D60A^*, and *cdgS^GGAAF^* using antibody against CdgS (indicated by arrow). For comparison of protein amounts loaded, samples were separated by electrophoresis and stained with Coomassie brilliant blue (CBB). (D) Analysis of the kinetics of c-di-GMP synthesis activity of CdgS and CdgS^D60A^. The top panel shows curve fitting to a Michaelis–Menten equation by nonlinear regression analysis, and the Lineweaver-Burk graphic method was used to calculate the enzyme kinetic parameters, V(max) and Km. Assays were performed using GTP as the substrate at various concentrations. The assays were performed thrice, and the average value and standard deviations are shown.

To investigate the role of c-di-GMP metabolic enzymes in cell size control, the cell morphology of individual in-frame markerless deletion mutants for each of the 16 genes of *Anabaena*, as previously reported (29), were analyzed. When observed under a microscope, only *ΔcdgS* was significantly affected in cell size while all others had a cell size comparable to that of the WT (Fig. 2A; Fig. S1 in the supplemental material), as already described (28). For the *ΔcdgS* mutant, cell length was 3.2 μm and cell width was 3.1 μm, which were reduced by 0.9 μm and 0.8 μm, respectively, compared to the WT strain. In terms of cell volume, the *ΔcdgS* mutant (19.1 ± 5.2 μm^3^) was only 37% to that of the WT (51.2 ± 9.7 μm^3^) (Table 1). Interestingly, the cell aspect ratio of the *ΔcdgS* mutant was maintained around 1.05, similar to that of the WT (Fig. 2B). These results indicated that the *ΔcdgS* mutant reduced cell length and cell width in a similar proportion, unlike the *yhjH* overexpression strain OE_CT_-*yhjH*, which reduced cell width more than cell length (aspect ratio 0.84 ± 0.16) (Fig. 1D). The effect on cell size in the *ΔcdgS* mutant could be complemented by the expression of a WT copy of *cdgS* on a replicative plasmid (strain C*cdgS*) (Fig. 2A and B). Taken together, among the 16 predicted c-di-GMP metabolic genes on the genome, *cdgS* is the major one involved in the regulation of cell size in *Anabaena* under the tested condition.

### The DGC activity of CdgS is required for cell size regulation.

CdgS has a C-terminal GGDEF domain that contains the conserved motif for DGC catalytic activity (29). To determine whether the cell size defects observed in *ΔcdgS* were caused by reduction of the intracellular c-di-GMP level or other functions of the protein, we generated a strain C*cdgS^GGAAF^*, similar to the complemented strain C*cdgS*, except that the gene *cdgS* was replaced by *cdgS*^GGAAF^ encoding a CdgS defective in c-di-GMP synthesis. We found that no phenotypic complementation of cell size occurred in C*cdgS^GGAAF^* (Fig. 2A and B, Table 1). Furthermore, we also replaced on the chromosome the WT copy of *cdgS* by *cdgS^GGAAF^*, and found that the so-generated strain had a similar phenotype as the *ΔcdgS* mutant (Fig. 2A and B, Table 1). Western blotting experiments confirmed that CdgS^GGAAF^ was expressed at a similar level as CdgS in the WT (Fig. 2C). From these observations, we concluded that the DGC activity of CdgS was required for cell size regulation in *Anabaena*.

To further determine that cell size regulation is dependent on the changes of c-di-GMP level caused by the DGC activity of CdgS, we checked the cell size parameters following the expression of *ydeH* encoding a c-di-GMP synthetase from E. coli under the control of the synthetic gene expression system inducible by theophylline and copper (29), in the *cdgS* mutant background (*ΔcdgS::ydeH*). As a control, a strain *ΔcdgS::ydeH^GGAAF^*, expressing an inactive form of YdeH, was also constructed. When grown in BG11 medium in the presence of 2 mM theophylline and 0.5 μM Cu^2+^, the cell size defects of the *ΔcdgS* mutant could be restored by the expression of YdeH, but not by that of the inactive form of YdeH^GGAAF^ (Fig. 2A and B, Table 1). This result, together with our previous finding on the reduction of the c-di-GMP level in the *ΔcdgS* mutant (29), indicated that the cell size effect of *ΔcdgS* was caused by a reduction of the c-di-GMP level.

### DGC activity of CdgS was regulated by its phosphorylation state at a conserved Asp residue.

**FIG 3:**
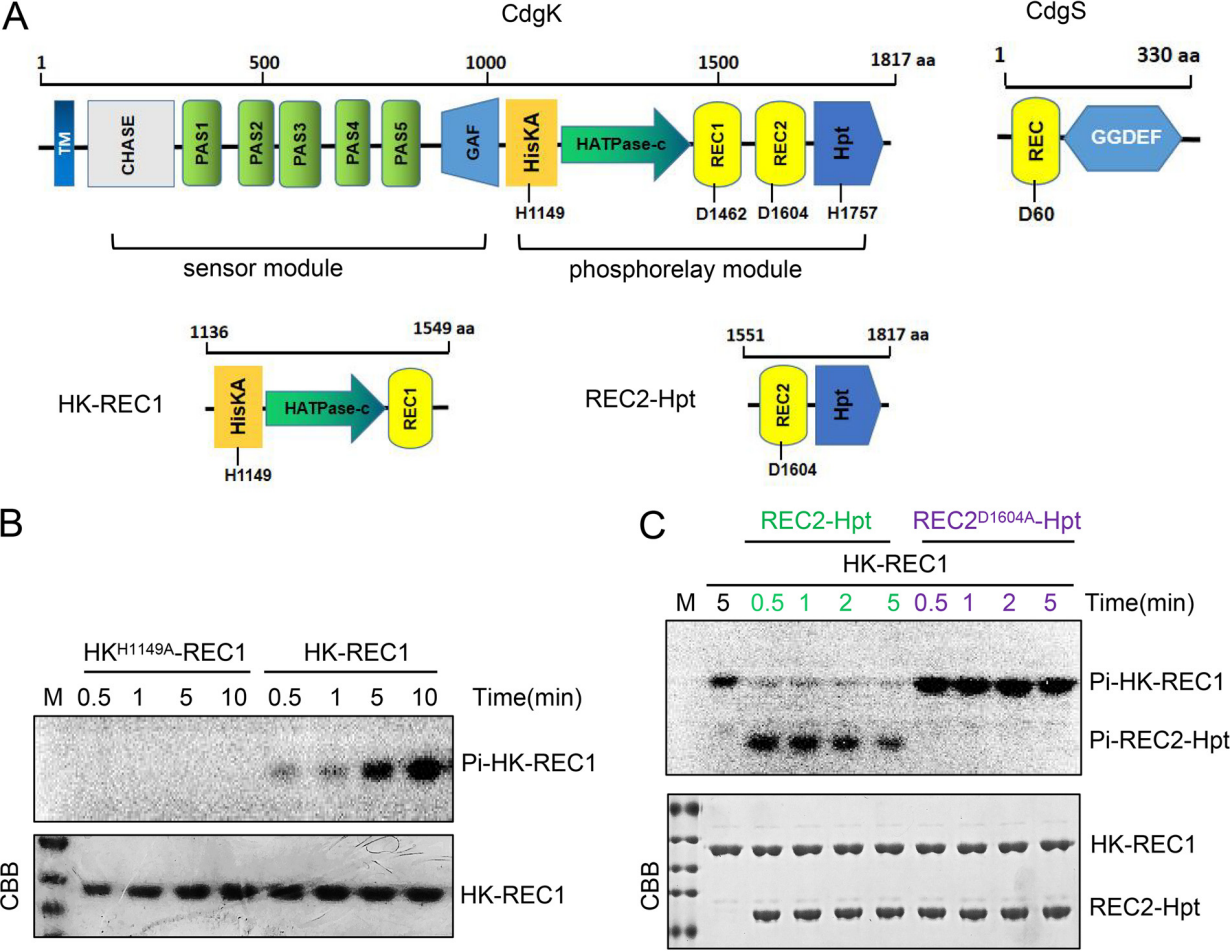
Phosphorylation assay of the histidine kinase CdgK *in vitro*. (A) Schematic representation of CdgK, CdgS, and the conserved residues involved in signal transduction through phosphorylation. TM, Transmembrane region; CHASE: Cyclases/Histidine kinases Associated Sensory Extracellular; PAS: Per-period circadian protein, Arnt-Ah receptor nuclear translocator protein, Sim-single-minded protein; GAF: Domain present in phytochromes and cGMP-specific phosphodiesterases; HisKA: dimerization and phosphoacceptor domain of histidine kinases; HATPase-c: Histidine kinase-like ATPases; REC: CheY-homologous receiver domain; Hpt: Histidine Phosphotransfer domain; GGDEF: diguanylate cyclase motif. The two truncated forms of CdgK, HK-REC1 and REC2-Hpt, are also shown. (B) Autophosphorylation of HK-REC1 and HK^H1149A^-REC1 polypeptides in the presence of [γ-32P]-ATP, followed by separation by using SDS/PAGE. Radioactive signals from the phosphorylated proteins were detected by autoradiogram (upper panel). The gel was stained with Coomassie brilliant blue (CBB, lower panel). (C) Transphosphorylation from HK-REC1 to REC2-Hpt. Autophosphorylation of HK-REC1 was enabled for 5 min prior to incubation with REC2-Hpt or REC2^D1604A^-Hpt.

A REC domain with a conserved Asp residue (Asp60) was found at the N-terminus of CdgS, which could be part of a phosphorelay system by accepting a phosphoryl group from the cognate HK (Fig. 3A, Fig. S2B). To investigate the role of Asp60, we generated a strain *cdgS^D60A^*, in which the Asp60 residue was substituted by an Ala residue at the native chromosomal locus in *Anabaena*. Phenotypic analysis indicated that the *cdgS^D60A^* mutant displayed similar cell size defects as the *ΔcdgS* mutant (Fig. 2A and B, Table 1). Western blotting results confirmed that CdgS^D60A^ was expressed in a similar level as the WT allele (Fig. 2C). These results strongly suggested that Asp60 played an essential role in the function of CdgS.

To further investigate the function of the conserved Asp60, we expressed and purified the recombinant CdgS and CdgS^D60A^ proteins from E. coli and first compared their properties by using size exclusion chromatography. As show in Fig. S3, CdgS^D60A^ presented a similar elution profile as CdgS, indicating that the D60A mutation did not change the oligomerization state of CdgS. Next, the DGC activity of this protein was then determined. Our results showed that CdgS^D60A^ could still synthesize c-di-GMP using GTP as the substrate (Fig. S3C). When the steady-state kinetic parameters for CdgS and CdgS^D60A^ were quantified under the same experimental conditions, the kinetic curves for c-di-GMP synthesis of CdgS and CdgS^D60A^ followed Michaelis–Menten kinetics (Fig. 2D). While the *Km* value for CdgS^D60A^ was similar to that of CdgS, the *Vmax* and *Kcat* values for CdgS^D60A^ were lower than those of CdgS. These results indicated that the D60A mutation did not change the substrate binding efficiency of CdgS, but inhibited the turnover efficiency. Indeed, the catalytic activity of CdgS, expressed by *Kcat*/*Km*, was 12.6 μM^−1^S^−1^, compared to 9.5 μM^−1^S^−1^ for CdgS^D60A^ (Fig. 2D, bottom). Taken together, our results suggest that phosphorylation of CdgS increases its DGC activity.

### Inactivation of *cdgK* encoding a histidine kinase led to a similar phenotype as *ΔcdgS*.

**FIG 4:**
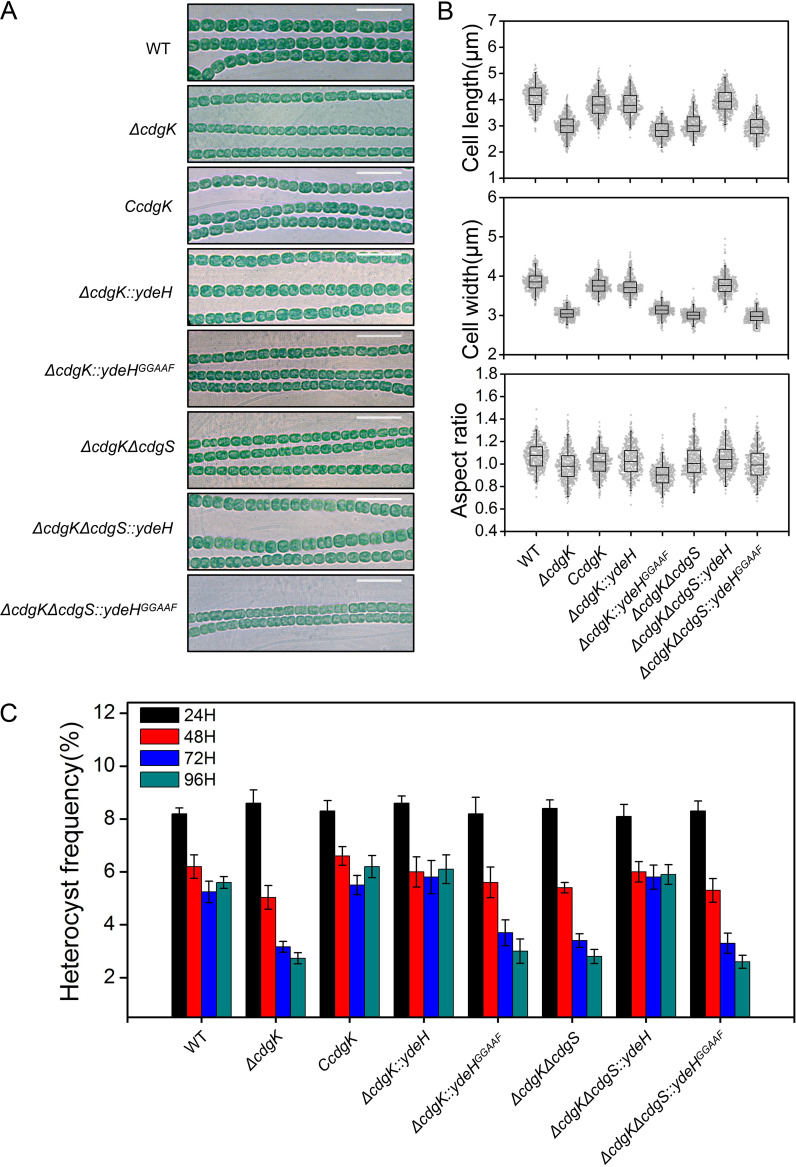
Inactivation of *cdgK* affects cell size and heterocyst development. (A) Microscopic observation of *Anabaena* filaments of different strains: WT, *ΔcdgK*, *CcdgK* (complementation of *ΔcdgK*), *ΔcdgK::ydeH*, *ΔcdgK::ydeH^GGAAF^*, *ΔcdgKΔcdgS*, *ΔcdgKΔcdgS::ydeH*, *ΔcdgKΔcdgS:: ydeH^GGAAF^*. Scale bars: 15 μm. (B) Statistic analysis of cell length, cell width, and aspect ratio based on images as shown in panel A. Five hundred cells of each strain were measured. (C) Heterocyst frequency of the indicated strains at different time points after nitrogen stepdown (24, 48, 72, and 96 h). All values of heterocyst frequency are shown as mean ± standard deviation, calculated using about 100 filaments.

Immediately upstream of *cdgS*, one open-reading frame, *all2875* (named *cdgK*) encoding a putative HK, was found. The two genes were cotranscribed and thus formed an operon (Fig. S4). To analyze the function of *cdgK*, we generated a markerless deletion mutant *ΔcdgK*. The *ΔcdgK* mutant reduced both its cell length (3.0 μm), its cell width (3.1 μm), and cell volume (22.6 ± 4.8 μm^3^) similarly as the *ΔcdgS* mutant (Fig. 4A and B, Table 1).

Previously, we (29) and others (28) reported that *cdgS* was involved in the regulation of the heterocyst pattern under diazotrophic conditions. We thus also checked the phenotype related to heterocyst differentiation in the *ΔcdgK* mutant. As shown in Fig. 4C, after 24 h of combined-nitrogen deprivation, heterocyst frequency of *ΔcdgK* was similar to that of WT, but it dropped to only 2.6% in comparison to 5.5% in the WT 96 h after induction. Therefore, the phenotypes of the *ΔcdgK* mutant were comparable to those of the *ΔcdgS* mutant in terms of both cell size and heterocyst frequency (Fig. 4). The defects of cell size and cell differentiation of the *ΔcdgK* mutant could be complemented by a replicative plasmid carrying a WT copy of *cdgK* under the control of its native promoter (strain C*cdgK*) (Fig. 4, Table 1). To investigate the relationship between *cdgK* and *cdgS*, we constructed a *ΔcdgKΔcdgS* double mutant and found that the double mutant displayed phenotypes similar to those of the *ΔcdgS* or *ΔcdgK* single mutant, including cell size reduction and lower heterocyst frequency (Fig. 4). To further confirm that the reduction of cell size or heterocyst frequency in the mutants was caused by a drop in the c-di-GMP level, we analyzed these phenotypes in a strain expressing *ydeH* in either the *ΔcdgK* single mutant or the *ΔcdgKΔcdgS* double mutant background, leading to strain *ΔcdgK::ydeH* or *ΔcdgKΔcdgS::ydeH*, respectively. The cell size and heterocyst frequency of these recombinant strains were restored to the WT level. In contrast, neither *ΔcdgK::ydeH^GGAAF^* nor *ΔcdgKΔcdgS::ydeH^GGAAF^* was complemented for the defects in cell size and cell differentiation (Fig. 4, Table 1). These results indicated that CdgK regulated cell size and heterocyst development by controlling the intracellular level of c-di-GMP.

### CdgK is a multidomain hybrid histidine kinase.

CdgK is predicted to be a membrane-anchored hybrid HK with a calculated molecular weight of 204.5 kDa (Fig. 3A). It has 12 domains likely located in the cytoplasm, which can be grouped into two modules: the sensor module containing the first 7 domains at the N-terminal, and the phosphorelay module containing the last 5 domains at the C-terminal (Fig. 3A). A transmembrane segment, a CHASE domain, five adjacent PAS domains, and a GAF domain are found at the N-terminal sensor module. The C-terminal phosphorelay module comprises a HisKA (DHp) domain with the conserved histidine residue (H^1149^) predicted as the primary phosphoryl group acceptor, an ATP-binding HATPase C (CA) domain, two putative REC domains with each containing a conserved Aspartic residue (D^1462^ and D^1604^, respectively), and a histidine phosphotransfer (Hpt) domain with the conserved histidine residue (H^1757^) predicted to be part of a phosphorelay system (Fig. 3A, Fig. S2).

To confirm that CdgK is indeed an HK, we checked its autophosphorylation activity *in vitro*. We tried to express the predicted cytoplasmic portion of CdgK, with different domain truncations in E. coli. After all assays, only the strep-tagged HK-REC1, which contained the HisKA, HATPaseC, and REC1 domains, could be expressed and purified with correct folding after purification (Fig. 3A, Fig. S5), while other recombinant polypeptides could not be expressed, were not soluble, or were not correctly folded. For the autophosphorylation test, HK-REC1 was incubated with [γ-^32^P]-ATP. As shown in Fig. 3B, the phosphorylated HK-REC1 was detected after 30 s of incubation, and the radioactive signal increased overtime. In contrast, when we incubated with [γ-^32^P]-ATP, the purified HK^H1149A^-REC1, a mutant with a substitution in the predicted phosphoacceptor site, no phosphorylated protein could be detected even after 10 min of incubation. These results confirmed that CdgK was an HK with autophosphorylation activity.

### Function of different domains of CdgK in the control of cell size.

**FIG 5:**
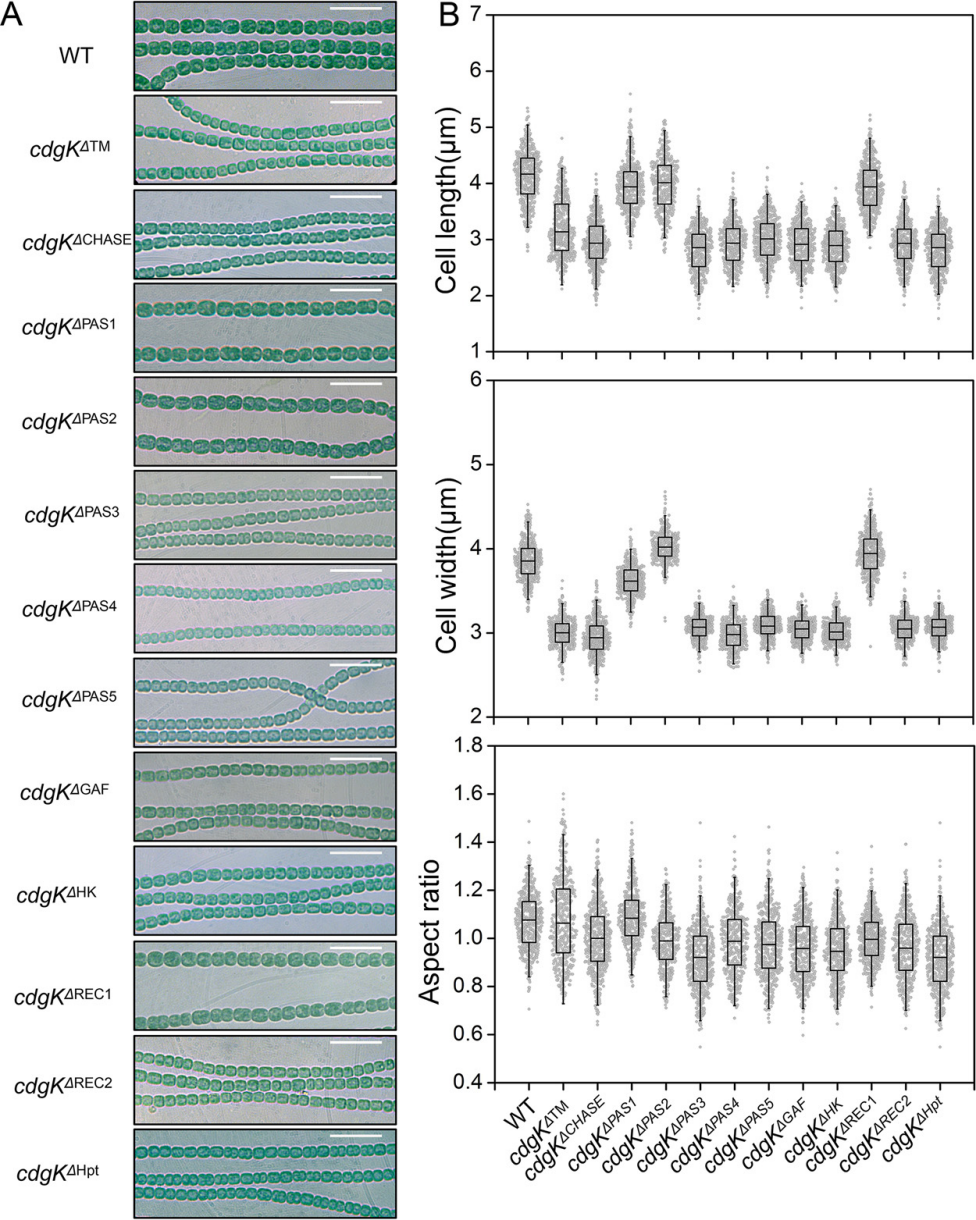
Requirement of different domains of CdgK in cell size control. (A) The micrographs of *cdgK* truncation mutants: *cdgK^ΔTM^* (deletion: 21 to 47 aa), *cdgK^ΔCHASE^* (deletion: 59 to 278 aa), *cdgK^ΔPAS1^* (deletion: 312 to 430 aa), *cdgK^ΔPAS2^* (deletion: 437 to 555 aa), *cdgK^ΔPAS3^* (deletion: 563 to 677 aa), *cdgK^ΔPAS4^* (deletion: 689 to 755 aa), *cdgK^ΔPAS5^* (deletion: 814 to 931 aa), *cdgK^ΔGAF^* (deletion: 946 to 1114 aa), *cdgK^ΔHK^* (deletion: 1130 to 1392 aa), *cdgK^ΔREC1^* (deletion: 1409 to 1523 aa), *cdgK^ΔREC2^* (deletion: 1548 to 1668 aa), and *cdgK^ΔHpt^* (deletion: 1682 to 1809 aa). Scale bars: 15 μm. (B) Statistic analysis of cell morphology based on microscopic images as shown in panel A. Five hundred cells of each strain were measured with triplicate data.

Since CdgK has two modules with 12 domains, we investigated the function of the different domains of each module. To study the role of CdgK domains in the phosphorelay module *in vivo*, we constructed mutant strains *cdgK^ΔHK^* (HisKA and HATPaseC domains deleted together), *cdgK^ΔREC1^*, *cdgK^ΔREC2^*, and *cdgK^ΔHpt^* with in-frame deletion of each corresponding domain, and analyzed their cell size and heterocyst frequency. The *cdgK^ΔREC1^* mutant did not show any particular phenotype in cell size and heterocyst differentiation, while the others, including *cdgK^ΔHK^*, *cdgK^ΔREC2^*. and *cdgK^ΔHpt^* mutants, all displayed a reduced cell length, cell width, cell volume, and heterocyst frequency (Fig. 5, Table 1, Table S4). This suggested that REC1 was a pseudo receiver domain and that the phosphoryl group from the HisKA domain could be transferred to the REC2 domain, and then to the Hpt domain. To confirm this possibility, a strep-tagged REC2-Hpt, which contained the REC2 and Hpt domains (Fig. 3A), was incubated with HK-REC1 in the presence of [γ-^32^P]-ATP. As shown in Fig. 3C, the phosphorylation level of HK-REC1 declined about 95% in the first 30 s, at the same time 95% of REC2-Hpt was phosphorylated. With time, the phosphorylation level of REC2-Hpt gradually decreased. Moreover, when the conserved Asp residue D1604 of REC2 was substituted by Ala, the mutant allele REC2^D1604^-Hpt failed to accept the phosphoryl group from HK-REC1 (Fig. 3C).

**FIG 6:**
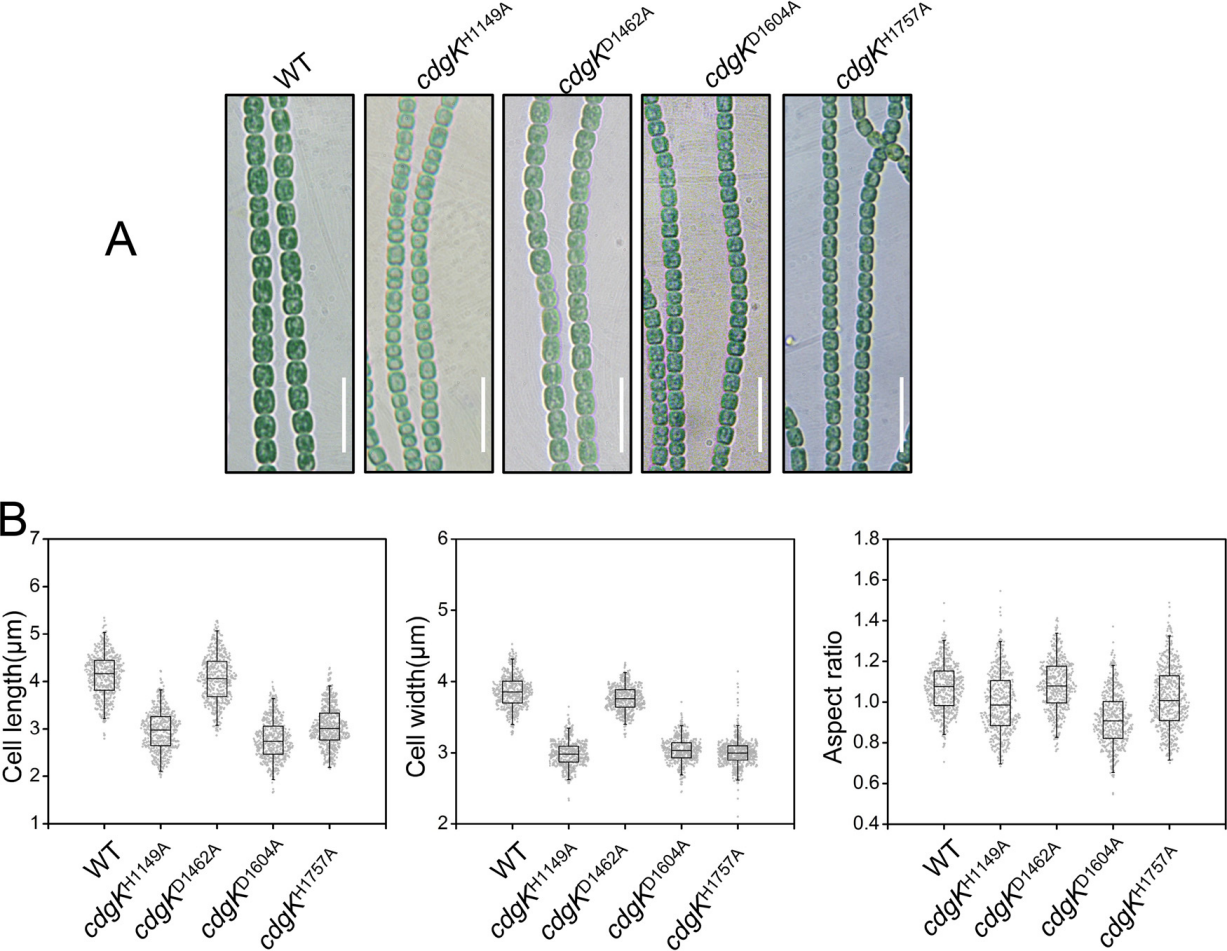
Effect of potential phosphorylation sites in cell size control. (A) The micrographs of WT, *cdgK^H1149A^*, *cdgK^D1462A^*, *cdgK^D1604A^*, and *cdgK^H1757A^* in BG11 medium. Scale bars: 15 μm. See Fig. 3A for key residues mutated here. (B) Statistic analysis of cell morphology. Five hundred cells of each strain were measured from triplicate data.

The results presented above indicated that CdgK possessed intramolecular phosphotransfer activity in the phosphorelay module. To further confirm the signaling route in CdgK, we constructed mutant strains with corresponding point mutations in which the conserved His residue or Asp residue of each domain in the phosphorelay module was changed to Ala, respectively (Fig. 6). Our results suggested that *cdgK^H1149A^*, *cdgK^D1604A^*, and *cdgK^H1757A^* mutants all display reduced cell size and heterocyst frequency as a *ΔcdgK* mutant, but *cdgK^D1462A^* presented no particular phenotype (Fig. 6, Table 1, Table S4). These results were consistent with the those obtained following truncation of the different domains (Fig. 5). All this genetic evidence indicated that CdgK and CdgS constituted a TCS system, and with the route of signal transduction in the following sequence: from H^1149^ within CdgK-HK, to D^1604^ within CdgK-REC2, then to H^1757^ within CdgK-Hpt, and finally to D^60^ within CdgS.

To investigate the role of different domains in the sensor module and the transmembrane segment of CdgK *in vivo*, we constructed the following mutants: *cdgK^ΔTM^*, *cdgK^ΔCHASE^*, *cdgK^ΔPAS1^*, *cdgK^ΔPAS2^*, *cdgK^ΔPAS3^*, *cdgK^ΔPAS4^*, *cdgK^ΔPAS5^*, and *cdgK^ΔGAF^*, each with in-frame deletion of the corresponding domain. Their cell size and heterocyst frequency were then analyzed (Fig. 5, Table 1, Table S4). The *cdgK^ΔTM^* mutant displayed similar cell size reduction to the *ΔcdgK* mutant (Fig. 5, Table 1), suggesting that the membrane anchoring of CdgK was required for its function. The cell size of *cdgK^ΔPAS1^* and *cdgK^ΔPAS2^* mutants were similar to that of the WT strain, while *cdgK^ΔCHASE^*, *cdgK^ΔPAS3^*, *cdgK^ΔPAS4^*, *cdgK^ΔPAS5^*, and *cdgK^ΔGAF^* mutants presented reduced cell size similarly as the *ΔcdgK* mutant (Fig. 5, Table 1). These results indicated that the CHASE, PAS3, PAS4, PAS5, and GAF domains may function as sensor modules for the activity of the HK or the transfer of the phosphoryl group, whereas PAS1 and PAS2 domains were unlikely to be involved in the regulation of cell size, or alternatively, PAS1 and PAS2 could play a negative regulation on cell size control.

## DISCUSSION

Despite the importance of c-di-GMP in regulating cell physiology in bacteria, the function of this second messenger is still poorly understood in cyanobacteria, particularly in heterocyst-forming, filamentous ones. Previously, it has been reported in *Anabaena* that CdgS acted as a DGC, and CdgSH had a prevailing PDE activity in spite of the presence of both DGC and PDE-related domains in the same protein (29). Genetic analyses revealed that CdgS and CdgSH controlled heterocyst frequency in *Anabaena* through their enzymatic activities of c-di-GMP metabolism (28, 29). In the present study, we provided evidence that the intracellular c-di-GMP level was important for cell size maintenance in *Anabaena*. As a fundamental physical trait characteristic of each bacterial species, cell size control has been extensively studied in model bacteria such as E. coli and B. subtilis (38). However, such studies have been very scarce for cyanobacteria, despite the ecological importance of these organisms. Previously, *cdgS* and the *mreBCD* operon were found to be involved in cell size regulation (28, 33). Our data presented here, obtained using various mutants of *cdgS* and *cdgK*, as well as strains with heterologous expression of E. coli enzymes for c-di-GMP synthesis or degradation, all indicated that a minimum threshold, or a local concentration, of intracellular c-di-GMP level was necessary for maintaining standard cell size (Fig. 1 and 2, Table 1). When c-di-GMP levels dropped following an inactivation of *cdgS*, or the expression of *yhjH*, cell size was reduced. However, in various strains, including the one expressing *ydeH* leading to an increase of c-di-GMP as shown previously (29), or those with deletion of the PDE domain, no changes in cell size were observed. Interestingly, both *ΔcdgK* and *ΔcdgS* mutants grow well as the WT under the tested conditions (Fig. S6), and cell width and cell length were reduced in a similar proportion by keeping the aspect ratio (Fig. 2 and 4). These results suggest that CdgS and CdgK constitute a major signaling pathway for cell size maintenance. Since nutrient regimes or light intensities have been reported to affect cell morphology in *Anabaena* (28, 33), it would be interesting to study if this regulatory pathway could integrate environmental signals for cell size control. Note that whereas the expression of *yhiH* in the OE_CT_-*yhjH* strain showed also a reduction in cell size, the reduction in cell width was more pronounced than that in cell length, thus leading to a change of the aspect ratio, in contrast to *ΔcdgS or ΔcdgK* mutants (Fig. 1 and 2, Fig. 3). One possible explanation was that the intracellular level of c-di-GMP in the OE_CT_-*yhjH* strain was slightly lower than that in *ΔcdgS*, as we reported previously (29). It may also imply that other c-di-GMP enzymes may have redundant functions in the control of cell size parameters.

**FIG 7:**
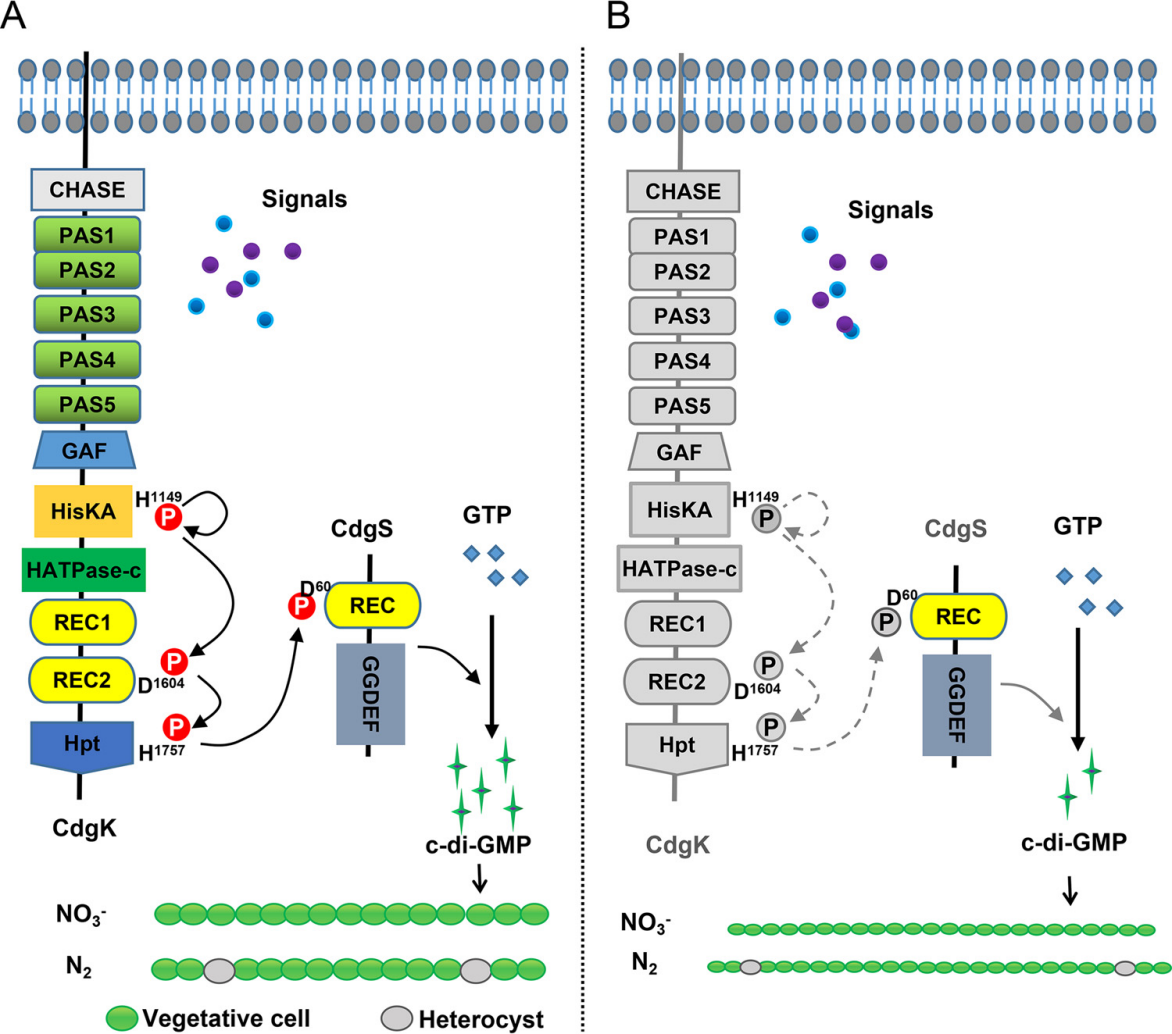
A model for the control of cell size and heterocyst development by the CdgK/CdgS signaling system in *Anabaena*. (A) CdgK senses the signal(s) by its sensor domains and gets autophosphorylated at the conserved histidine residue of the HisKA domain. The phosphate group is then transferred sequentially to the conserved aspartic acid residue of the RCE2 domain, the conserved histidine of the Hpt domain, and the aspartic acid residue of the REC domain of CdgS. The catalytic activity of CdgS was enhanced following phosphorylation, to maintain c-di-GMP above a threshold necessary for the control of cell size and heterocyst frequency as found in the WT. (B) Following inactivation of any of the key signaling pathway, CdgS activity is limited so that the intracellular c-di-GMP level decreases below the threshold, leading to a reduction of cell size as well as heterocyst frequency.

Our data indicated that the cell size control is mediated by the c-di-GMP synthetase activity of CdgS since its GGDEF domain is required for this function. In addition, CdgS is an RR with a conserved Asp residue. This residue is necessary for cell size control, and the DGC activity is reduced when this conserved residue is replaced by an Ala residue. These results imply that the phosphorylation status of CdgS modulates the level of c-di-GMP in the cells, and that the activity of CdgS could be controlled by a histidine kinase. We provided evidence that CdgK acted as the cognate histidine kinase for CdgS and the two proteins constituted a classical two-component signaling system as predicated (Fig. 3A). First, the two corresponding genes were cotranscribed and thus formed an operon (Fig. S4). Second, loss of *cdgK* or *cdgS*, or both genes together, led to similar phenotypes (Fig. 1, Fig. 4). CdgK was a large protein with a molecular weight of 204.5 kDa and a complex domain structure, which limited our ability to obtain the full-length protein for all *in vitro* biochemical characterization. Nevertheless, we were able to demonstrate the His kinase activities of CdgK by confirming its autophosphorylation and the intramolecular phosphotransfer activities (Fig. 3). Mutations of key residues and domains of CdgK and CdgS also indicated the existence of a phosphorelay system with the CdgK-CdgS system (Fig. 5 and 6). Based on these findings, we propose a model for the regulatory function of the system (Fig. 7). CdgK senses an unknown signal and phosphorylates CdgS. The phosphorylated form of CdgS (CdgS~P) then promotes its DGC activity and increases the c-di-GMP level in the cells, necessary to maintain cell size and heterocyst frequency (Fig. 7).

The functions of the CdgK-CdgS system in cell size control and heterocyst development are always linked. Indeed, whenever cell size reduction was observed in a mutant, a decrease of heterocyst frequency at the maintenance step was found. In all the complementation assays, both phenotypes could be recovered together. One possible explanation is that the reduction of cell size may affect intercellular communication or increase the relative concentrations of the inhibitory signals for heterocyst differentiation produced from source cells, leading to a diffusion over a distance longer than in the WT. These hypotheses could be tested experimentally in the future.

The TM domain at the N-terminal of CdgK is indispensable for cell size control (Fig. 5, Table 1). This result, together with the presence of multiple sensor domains in the cytoplasm, suggests that CdgK could be activated by both extracellular and intracellular signals (Fig. 3 and 5). It was recently reported that PAS2 in CdgK binds to FMN (flavin mononucleotide), suggesting that PAS2 could be a blue-light sensor (39). This hypothesis has yet to be confirmed by physiological and genetic studies. Therefore, the identification of the signals that regulate cell size control through the CdgK-CdgS system remains a challenge. Our studies presented here open an interesting perspective for exploring the molecular mechanism for the determination of cell size parameters, as well as their modulation according to environmental conditions.

## MATERIALS AND METHODS

### Bacteria strains, media, and growth conditions.

*Anabaena* sp. strain PCC 7120 and its derivatives were grown at 30°C in BG11 medium with shaking at 180 rpm and continuously illuminated with 30 μmol m^−2^ s^−1^ of light. For heterocyst induction, cells were grown to logarithmic phase in BG11, followed by washing and transference to BG11_0_ (BG11 without nitrate) medium as described (40). For induction of genes under the control of the artificial CT promoter (41), cells were grown first in copper-free BG11 medium to logarithmic phase, and then transferred to fresh BG11 medium containing 0.5 μM copper and 2 mM theophylline. Neomycin (25 μg/mL), streptomycin (2.5 μg/mL), or spectinomycin (5 μg/mL) was provided in the culture as needed. All strains used in this study are listed in Table S1.

### Mutant construction and DNA manipulation.

Markerless deletion mutants and strains with different point mutations were created by the genome editing technique based on CRISPR-Cpf1 as described (42). The plasmids, which contain the artificial CT promoter for gene overexpression by addition of inducers (copper and theophylline), were constructed based on the PCT vector (29, 41). To construct plasmids used for protein expression in E. coli, the corresponding DNA fragment was amplified and subcloned into the pHTwinStrep (pHTS) vector (a derivative from the protein expression vector pET28a), which contains a region encoding two tandem Strep-tags that can be fused to the C-terminus of a protein of interest.

The procedure of conjugation was similar to that described previously (43, 44). Mutants with a point mutation were created by two steps. Briefly, the gene to be modified was first substituted by a *gfp* coding sequence *in situ* with the corresponding plasmid by conjugation, forming a transcriptional fusion strain. Then a plasmid that contained a gene allele with point mutation was introduced to replace *gfp* following homologous recombination. The primers and plasmids used in this work are listed in Table S2 and Table S3, respectively.

### Protein domains, protein expression and purification, Western blotting, and diguanylate cyclase activity assay.

Protein domains were analyzed by using SMART (http://smart.embl-heidelberg.de/). Escherichia coli BL21(DE3), which contains plasmids based on pHTwinStrep, was cultured in LB with 50 μg mL^−1^ kanamycin at 37°C to an OD_600_ at 0.5 to 0.8 (exponential growth phase, measured using a spectrophotometer of UV-5200 from METASH, Shanghai). Protein expression was induced by adding 0.5 mM isopropyl-β-D-1-thiogalactopyranoside (IPTG) and cultured overnight at 16°C. The cells were collected by centrifugation at 8,000 × *g* for 5 min, resuspended with a lysis buffer (50 mM Tris-HCl, pH 8.0, 0.5 M NaCl), and lysed using a French press. The cell debris was removed by centrifuging at 12,000 × *g* at 4°C for 35 min. The supernatant containing the soluble proteins was collected and mixed with preequilibrated Strep-Tactin XT Superflow resin (IBA) for 1 h at 4°C. The resin was then collected by filtration and extensively washed with buffer W (50 mM Tris-HCl pH 8.0, 0.2 M NaCl). The proteins were subsequently eluted with elution buffer (buffer W containing 50 mM biotin), concentrated (Amicon Ultracel-10K, Millipore), and analyzed by size exclusion chromatography (SEC) using a Superdex 200 Increase 10/300 GL column (GE Healthcare) that was equilibrated with buffer W. Fractions containing the recombinant protein were pooled, concentrated again, and stored at −80°C. The purified protein was examined using 10% sodium dodecyl sulfate polyacrylamide gel electrophoresis (SDS-PAGE) to verify the molecular weight and purity. The protein concentration was determined by Bradford assay (45). Western blotting was carried out using a procedure as described (46). The DGC activity assay was analyzed as previously described (29, 47).

### *In vitro* autophosphorylation and transphosphorylation assays.

To detect the kinase activity of CdgK, purified HK-REC1 or HK^H1149A^-REC1 (16 μM) were incubated at 30°C with 70 kBqi [γ-^32^P]-ATP (PerkinElmer, USA) in a reaction buffer (50 mM HEPEs, pH 8.0, 50 mM KCl, 15 mM MgCl_2_, and 1 mM DTT). To detect phosphotransfer, HK-REC1 (16 μM), REC2-Hpt (26 μM), HK-REC1 (16 μM), or REC2^D1604^-Hpt (24 μM) was incubated, respectively, with 70 kBqi [γ-^32^P]-ATP under the same conditions as above. The reactions were terminated with 4×SDS-PAGE loading buffer at the indicated incubation time. The samples were heated at 90°C for 3 min and then analyzed by using a 10% SDS-PAGE. Phosphorylation signals were detected by autoradiography.

### Microscopy and statistical analysis.

Microscopic images were captured by using a SDPTOP EX30 microscope and processed using ImageJ without deconvolution as described (46). For cell size analysis, microscopic images were taken when optical density of a culture reached 0.3 to 0.5 at OD_750_ (exponential growth phase). Five hundred cells were randomly selected from the filaments in three biological replicates. The cell length and width of each strain were measured by ImageJ. The aspect ratio was estimated by cell length divided by cell width. The shape of *Anabaena* cells was assumed to be a cylinder; thus, the cell volume calculation equation is V = π × (1/2 cell width) × (1/2 cell width) × cell length. All cell volume values are shown as mean ± standard deviation, calculated using the results for 500 cells. Statistical tests and plotting of data were performed with Origin 8.0. For analysis of heterocyst frequency and percentage, microscopic images were taken at the indicated time points with culture incubated under combined-nitrogen deprivation conditions. Heterocyst frequency was calculated by determining the number of heterocysts in 2,000 cells from about 100 filaments randomly selected from three parallel cultures. All values are showed as mean ± standard deviation.
