# Erratum for Sun et al., “Control of Cell Size by c-di-GMP Requires a Two-Component Signaling System in the Cyanobacterium *Anabaena* sp. Strain PCC 7120”

**DOI:** 10.1128/spectrum.00155-24

**Published:** 2024-02-08

**Authors:** Qing-Xue Sun, Min Huang, Ju-Yuan Zhang, Xiaoli Zeng, Cheng-Cai Zhang

Volume 11, no. 1, e04228-22, 2023, https://doi.org/10.1128/spectrum.04228-22. Qing-Xue Sun’s first name was spelled incorrectly in the article. The name should appear as in the byline of this erratum.

