# Supplementary material for: Control of Cell Size by c-di-GMP Requires a Two-Component Signaling System in the Cyanobacterium Anabaena sp. Strain PCC 7120
Source: Microbiol Spectr. 2023 Jan 10;11(1):e04228-22. doi: 10.1128/spectrum.04228-22 (PMC9927289; doi:10.1128/spectrum.04228-22)
Supplement: Supplemental file 5 — Fig. S1-S6. Download spectrum.04228-22-s0005.pdf, PDF file, 1.2 MB [file spectrum.04228-22-s0005.pdf]

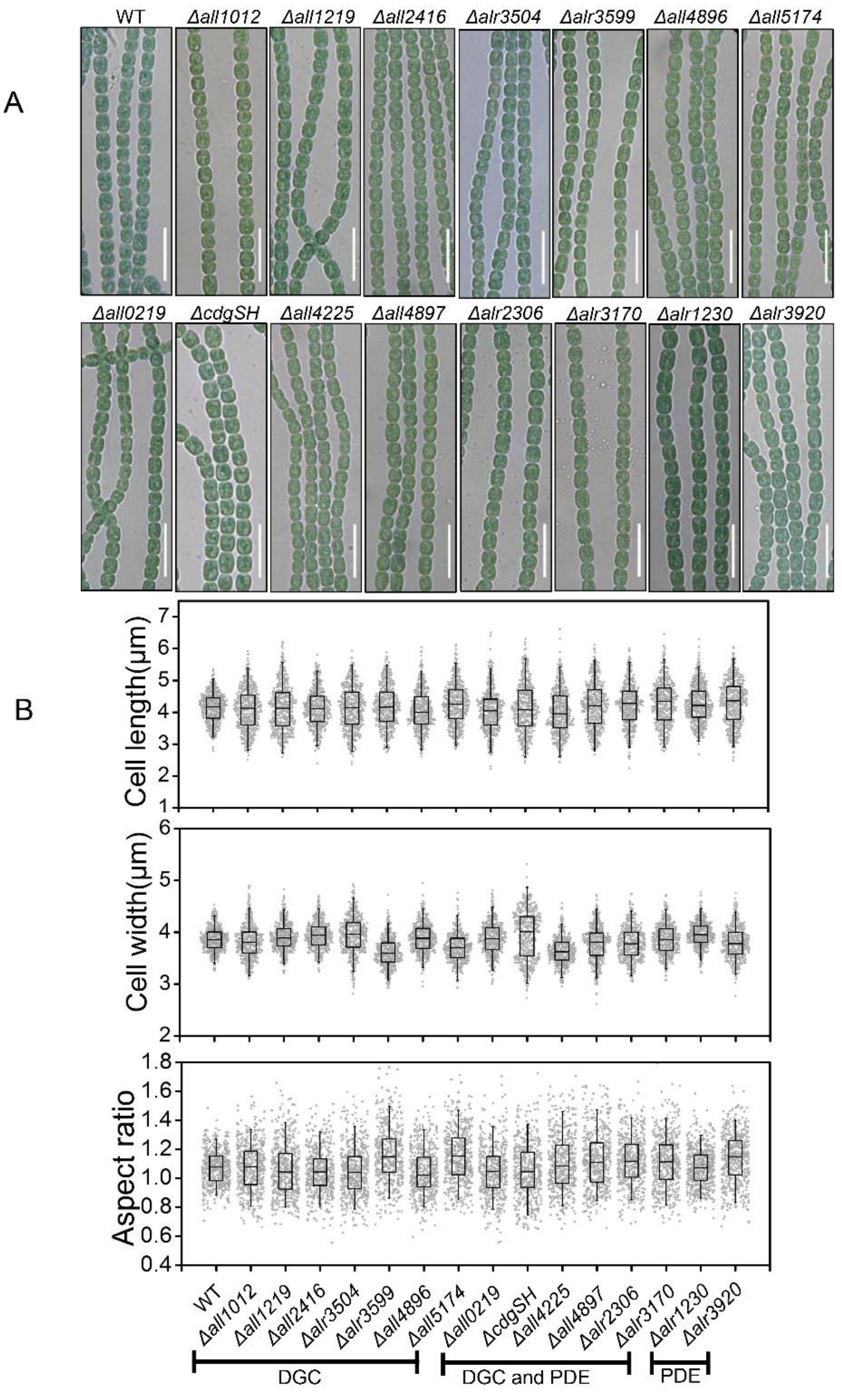

FIG S1 Inactivation of each of the 15 c-di-GMP metabolic genes in *Anabaena* did not affect the cell size. (A) Micrographs of filaments of the WT and different mutants. Scale bars: 15 μm. (B) Statistic analysis of cell length, cell width and the aspect ratio of the cells based on images in panel A. 500 cells of each strain were measured. DGC, diguanylate cyclase; PDE, phosphodiesterase.

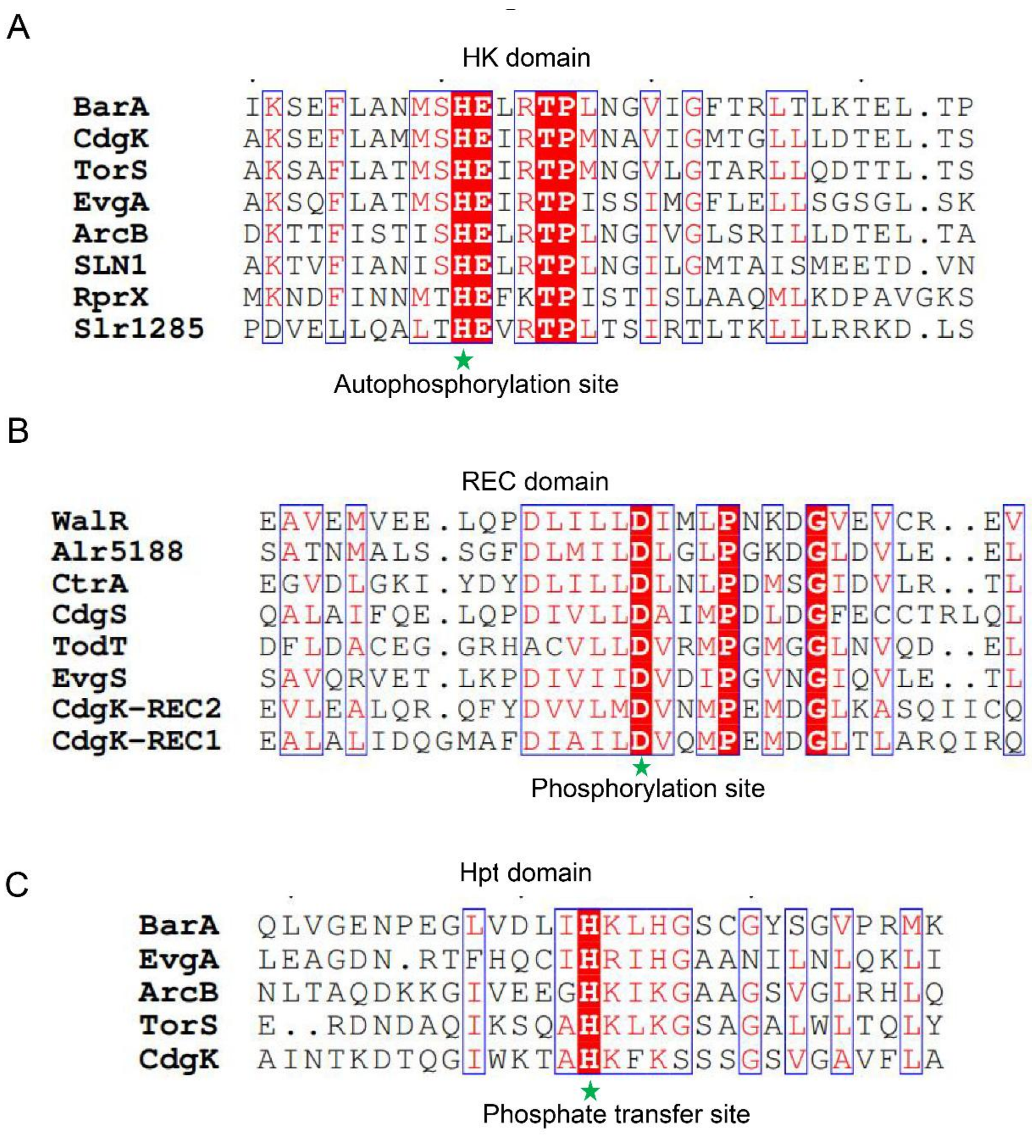

FIG S2 Sequence alignment of major domains in the CdgK-CdgS signaling system. The regions, which contain the conserved phosphorylation sites, were presented. (A) Sequence alignment of HK domains in CdgK and representative histidine kinases. Green asterisk indicates the autophosphorylation site. (B) Alignment of REC domains in CdgK, CdgS and representative response regulators. Green asterisk indicates the conserved aspartic acid residue for phosphorylation. (C) Sequence alignment of the conserved Hpt domains in CdgK and representative proteins. Green asterisk indicates the histidine residue for phosphorylation. ClustalX and ESPript 3.X were used for sequence alignment. All homologous domains of proteins were retrieved from NCBI (https://www.ncbi.nlm.nih.gov/). BarA (Genebank: APC52989.1); TorS (GenBank: AAF96608.1); EvgA (Genebank: BAA16241.1); ArcB (Genebank: CAA37397.1); LSN1 (Genebank: AJR37544.1); RprX

(Genebank: AAB26173.1); Slr1285 (Genebank: WP_010872912.1); WalR (Genebank: NP_391921); Alr5188 (Genebank: WP_010999312.1); CtrA (Genebank: WP_010920871.1); TodT (Genebank: APC70898.1); EvgS (Genebank: BAA16240.1).

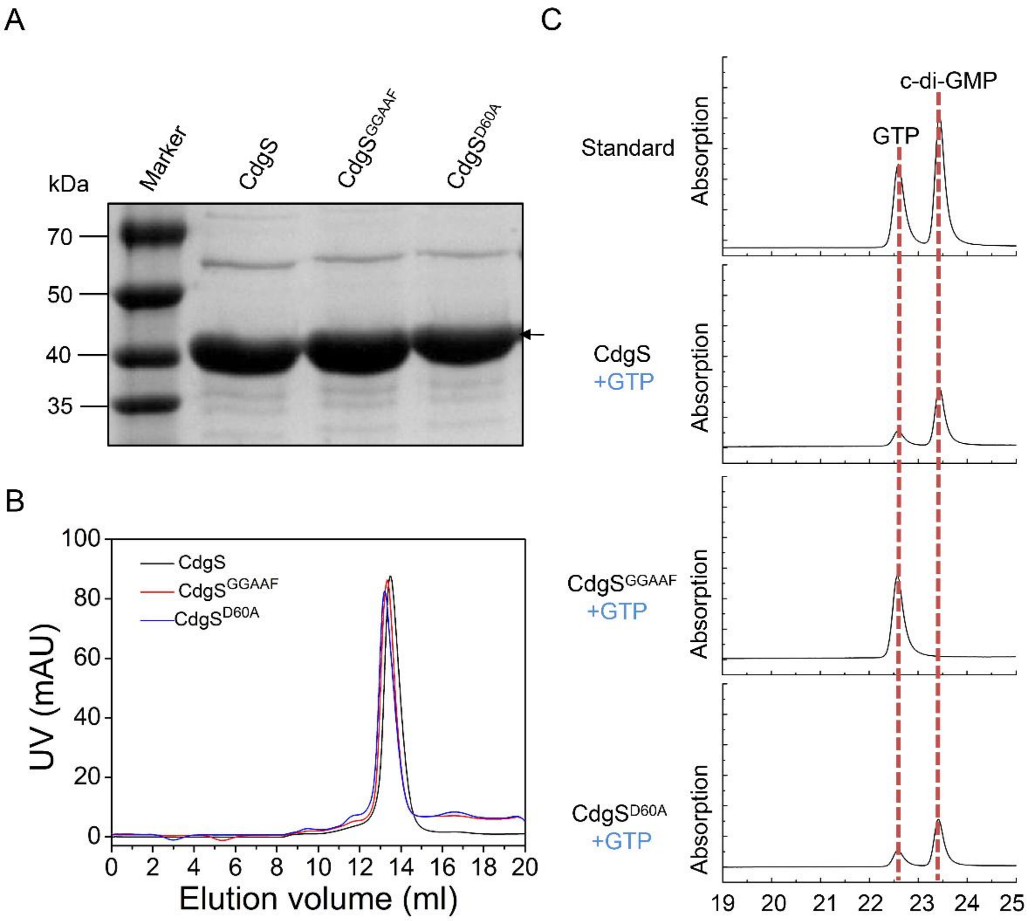

FIG S3 Mutation of the conserved D60 residue of CdgS affects its DGC activity. (A) SDS-PAGE analysis of purified proteins (indicated by arrow) for DGC activity assay. (B) Size exclusion chromatography analysis of CdgS, CdgS^{D60A} and CdgS^{GGAAF}. The elution peaks of these proteins are around 13.5 ml. (C) The DGC activities of CdgS, CdgS^{D60A} and CdgS^{GGAAF}, evaluated by the reaction product of c-di-GMP. All reaction products are assessed by the retention time in HPLC compared to the standards. The top panel shows the retention time of the standard nucleotides, including c-di-GMP and GTP. The protein used for DGC activity assay was indicated at the left of the chromatograms and the substrate for reaction (GTP) was indicated under the name of each protein.

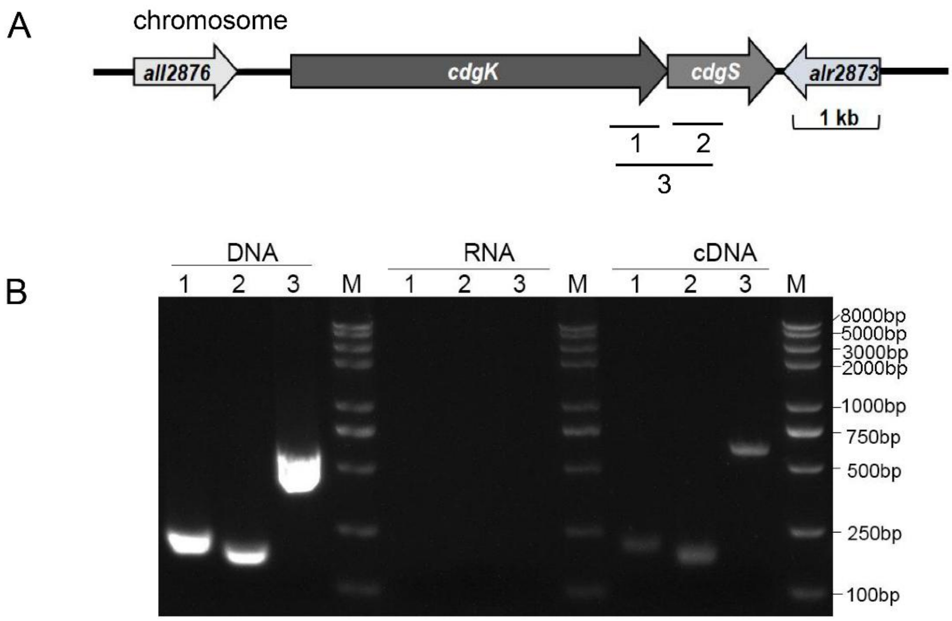

FIG S4 Co-transcription of *cdgK* and *cdgS*. (A) A schematic drawing of the *cdgK* and *cdgS* locus. Line 1 represents PCR fragment of *cdgK*, amplified with primers cr-cdgKR5200R and PcdgKR5454; line 2 represents PCR fragment of *cdgS*, amplified with primers PcdgSF1a and PcdgSR191; line 3 represents PCR fragment overlapping *cdgK* and *cdgS,* amplified with primers PcdgKF5005 and PcdgSR191. See Table S3 for details of the primers. (B). RT-PCR analysis of *cdgK* and *cdgS*. Total RNA was extracted using a Plant Total RNA Isolation Kit and reverse transcribed to cDNA for PCR analysis. Genomic DNA was used as a positive control, and RNA served as a negative control. M: DNA marker.

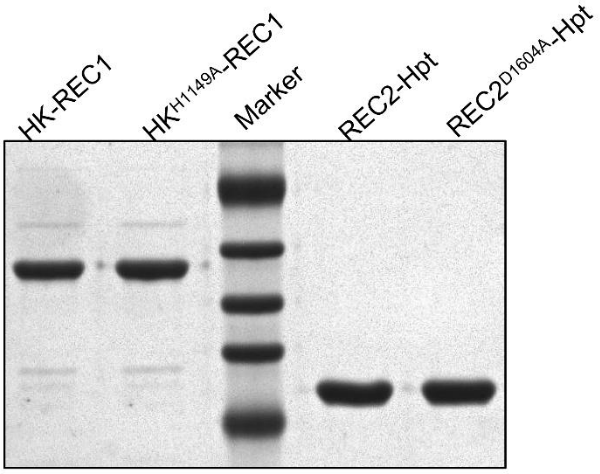

FIG S5 SDS-PAGE analysis of purified proteins used for autophosphorylation and intramolecular transphosphorylation assay.

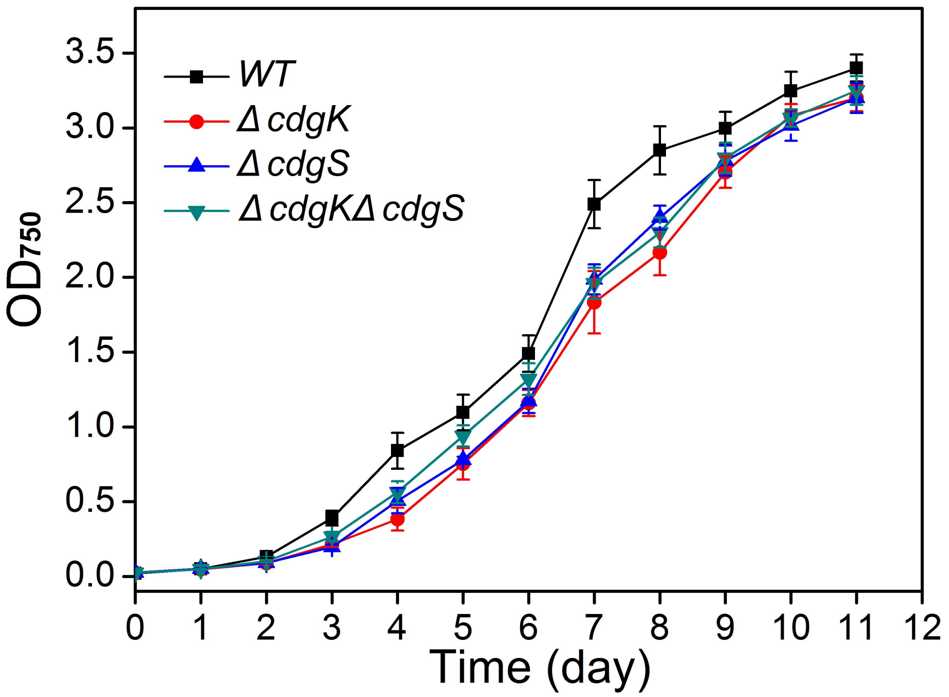

FIG S6 Growth of WT, *ΔcdgK, ΔcdgS* and *ΔcdgKΔcdgS* strains in BG11 medium. All values are shown as mean ± standard deviation, calculated from triplicate data.
